# Three-dimensional printed titanium mesh combined with iliac cancellous bone in the reconstruction of mandibular defects secondary to ameloblastoma resection

**DOI:** 10.1186/s12903-023-03386-0

**Published:** 2023-09-20

**Authors:** Zhiyang Zhao, Shunyao Shen, Meng Li, Guofang Shen, Guanrong Ding, Hongbo Yu

**Affiliations:** 1 Department of Oral and Craniomaxillofacial Surgery, Shanghai Ninth People’s Hospital, College of Stomatology, National Center for Stomatology, Shanghai Key Laboratory of Stomatology, Shanghai Jiao Tong University School of Medicine, Shanghai Jiao Tong University, National Clinical Research Center for Oral Diseases, Shanghai Research Institute of Stomatology, Shanghai, China; 2 Department of Radiology, Shanghai Ninth People’s Hospital, Shanghai Jiao Tong University, Shanghai, China

**Keywords:** Virtual surgical planning, Mandible reconstruction,3D-printing, Iliac cancellous bone, Surgical accuracy

## Abstract

**Background:**

The reconstruction of large mandibular defects is a challenge, and free vascularized bone flaps are most commonly used. However, the precision and symmetry of this repair are deficient, and patients have a risk of vascular embolism, flap necrosis, and donor site complications. Therefore, to explore an ideal alternative in mandibular reconstruction with high surgical accuracy and low complications is indispensable.

**Methods:**

Seven patients with recurrent or large-scope ameloblastoma were enrolled in this study. All patients were provided with a fully digital treatment plan, including the design of osteotomy lines, surgical guides, and three-dimensional printed titanium mesh for implantation. With the assistance of surgical guide, ameloblastomas were resected, and custom 3D printed titanium mesh combined with posterior iliac bone harvest was used in mandibular reconstruction. A comparison was made between the discrepant surgical outcomes and the intended surgical plan, as well as the average three-dimensional deviation of the mandible before and after the surgery. At the same time, the resorption rate of the implanted bone was evaluated.

**Results:**

All patients completed the fully digital treatment process successfully without severe complications. Image fusion showed that the postoperative contour of the mandible was basically consistent with surgical planning, except for a slight increase in the inferior border of the affected side. The mean three-dimensional deviation of the mandible between the preoperative and postoperative periods was 0.78 ± 0.41 mm. The mean error between the intraoperative bone volume and the digital planning bone volume was 2.44%±2.10%. Furthermore, the bone resorption rates of the harvested graft 6 months later were 32.15%±6.95%.

**Conclusions:**

The use of digital surgical planning and 3D-printed templates can assist surgeons in performing surgery precisely, and the 3D-printed titanium mesh implant can improve the patient’s facial symmetry. 3D printed titanium mesh combined with posterior iliac cancellous bone graft can be regarded as an ideal alternative in extensive mandibular reconstruction.

## Background

Ameloblastoma (AM) is an infrequent benign odontogenic tumor of the jaw, representing approximately 1% of all tumors located in the cephalic and cervical regions [1, 2]. This tumor predominantly manifests in mandibular body (constituting nearly 80%), specifically in molar and ramus anatomical regions. When confronted with extensive or multifocal recurrences, segmental mandibular resection becomes a requisite therapeutic modality The gold-standard treatment is not without risks, including vascular issues from compromise to flap necrosis. Post-op challenges also include facial asymmetry, dental rehab complexities, and flap failure risks. Donor sites may have complications like functional deficits and chronic pain. [3–5].

Virtual Surgical Planning(VSP) can simulate intraoperative operations, while avoiding important anatomical structures such as nerves, and simulating osteotomies and bone block movement, thus providing more efficient and predictable reconstruction results [6]. Three-dimensional (3D) printing technology further complements the prowess of VSP. As a result, the positioning of the moved bone block becomes notably more precise, which in turn improves both post-surgical bone block stability and overall surgical accuracy [7, 8]. In addition, the posterior iliac cancellous bone is commonly used in maxillofacial reconstruction [9]. It can provide enough amounts of bone while also preserving the outer contours of the iliac bone. Moreover, compared to those who underwent fibula or iliac bone harvesting, patients who receive posterior iliac cancellous bone grafts generally experience less severe complications at the donor site [10, 11].

For these reasons, this study aimed to explore the feasibility of 3D printed titanium mesh combined with a posterior iliac cancellous bone to repair mandibular defects caused by ameloblastoma, and surgical accuracy and long-term osteogenesis were evaluated.

## Materials and methods

### Patients

Seven patients with large scope or recurrent mandibular ameloblastomas were enrolled in this study. All patients (4 males and 3 females; mean age 38.86 years old) underwent ameloblastoma resection and sequential mandibular reconstruction with 3D printed titanium mesh and posterior iliac cancellous bone grafts. This study was approved by the Ethics Committee of the Ninth People’s Hospital (SH9H-2021-T65-1), and informed consent was obtained.

### Surgical planning

**Fig. 1:**
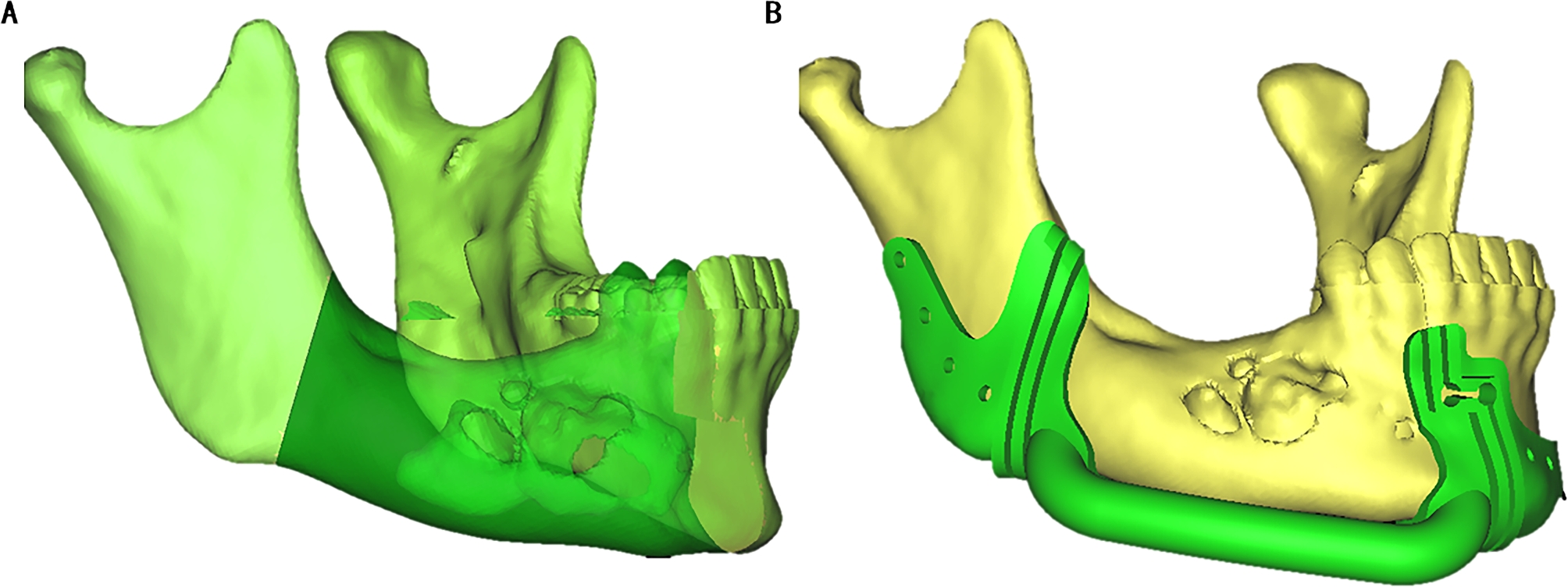
A clear view of the range of mandibular ameloblastoma (A), and the osteotomy templates (B)

For each case, clinical information was collected, including CT scans and dental plaster models. The CT scans had a pixel size of 0.45 mm x 0.45 mm, slice intervals of 1.25 mm, and a resolution of 512 × 512 × 231 (LightSpeed Ultra 16 spiral CT machine, GE Company, USA). The scan data was imported into ProPlan CMF 3.0 software (Materialise, Leuven, Belgium). The scope of ameloblastoma and osteotomy lines were defined in the horizontal, sagittal, coronal plane, and 3D reconstruction models (Fig. 1A). To ensure the precision of the procedure, the osteotomy templates were designed based on the location of the osteotomy line (Fig. 1B).

**Fig. 2:**
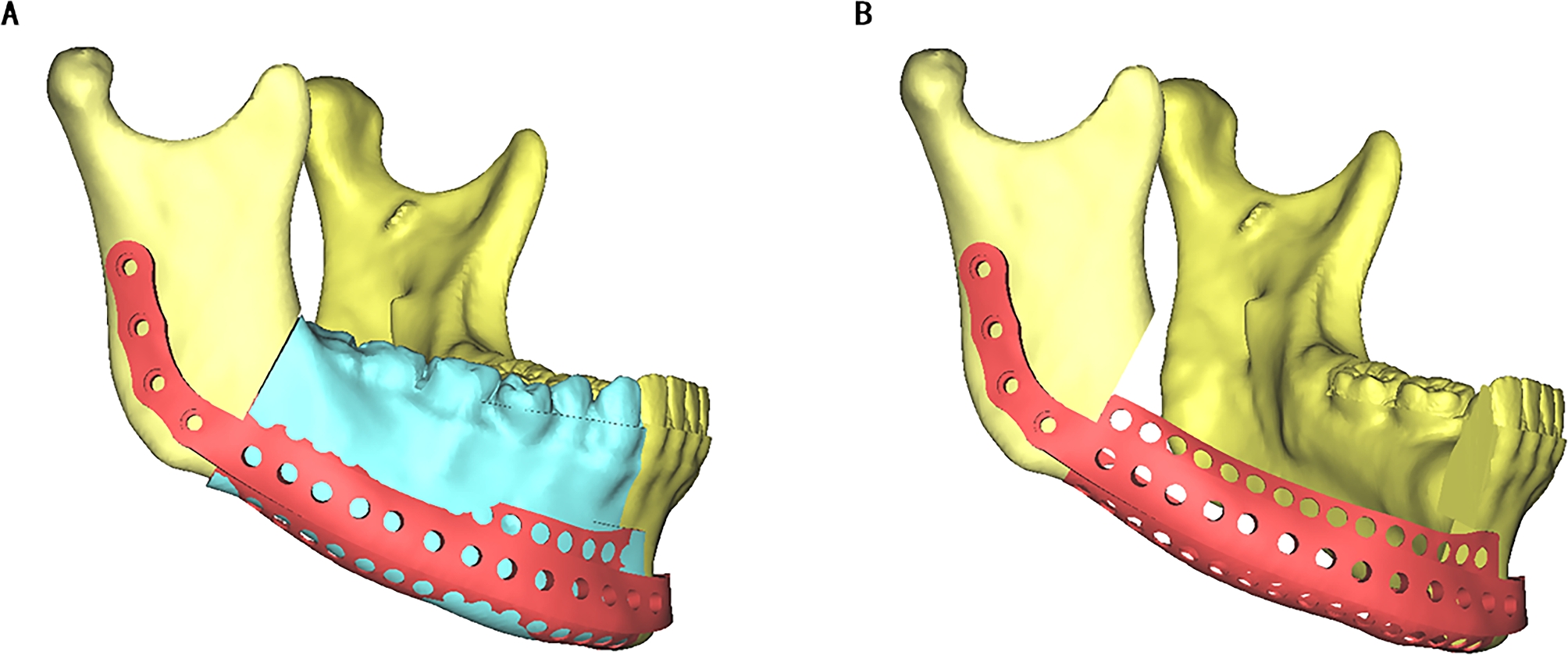
The 3D titanium mesh reconstruction model of the mandible. (A) The contour of the affected side of the mandible (blue) was mirrored from the unaffected side. (B) The 3D titanium mesh reconstruction model of the mandible was designed

Frankfort horizontal plane (FH), which was formed by connecting bilateral porions (P) and the left orbital point (OrL), was defined as the reference plane. Then the median sagittal plane (SP) was defined by the points of Sella (S), Nasion (N), and perpendicular to the FH in the patient’s 3D reconstruction model. To reconstruct the defect, the median sagittal plane was used as a reference plane. Normal anatomic structures and the contour of the target area were mirrored from the unaffected side. Thus, the normal contour of the affected area was ascertained (Fig. 2A). A virtual model of the titanium mesh restoration was designed based on the mirrored mandibular contour. (Fig. 2B).

**Fig. 3:**
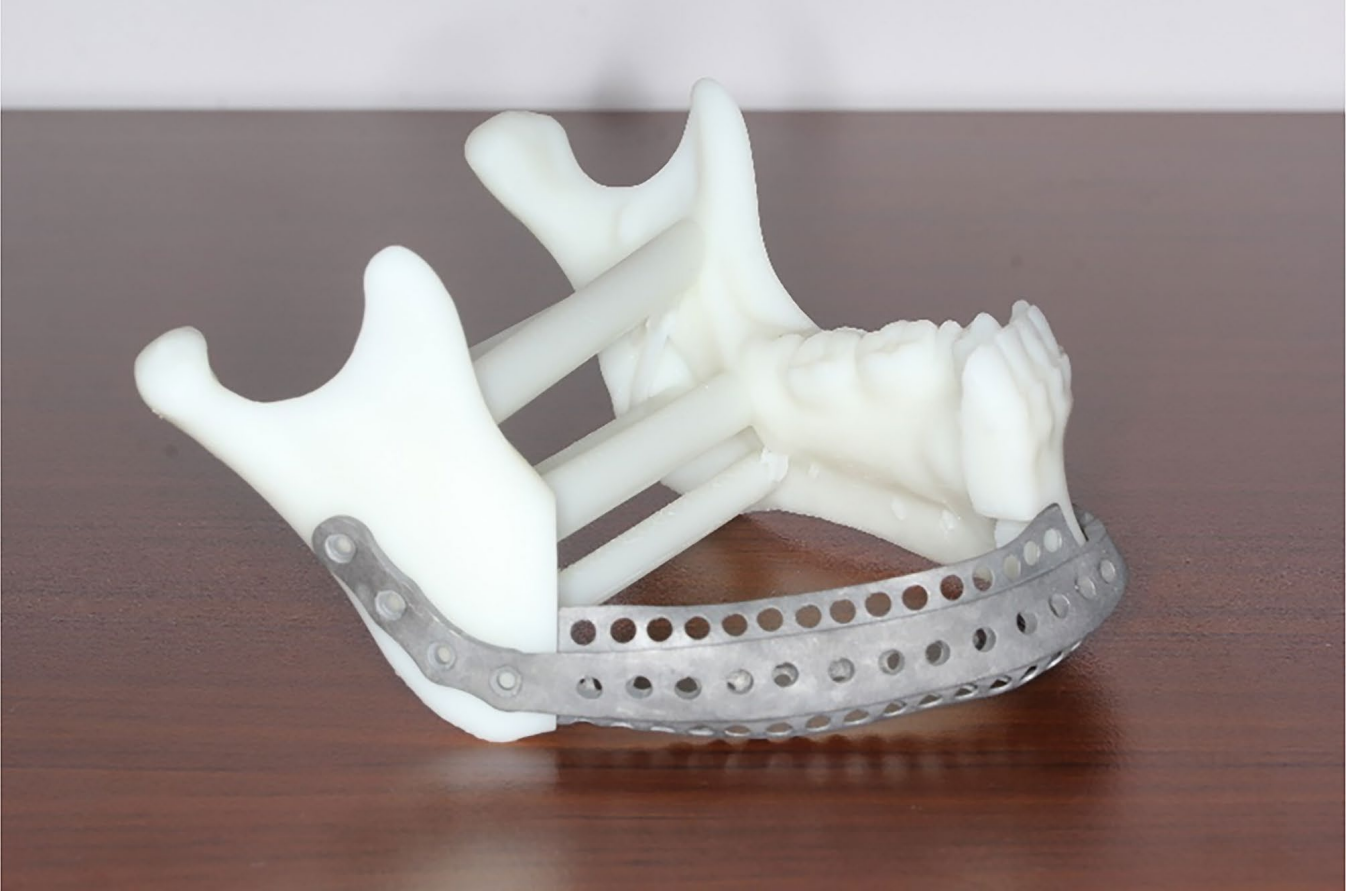
All models and templates were printed on a 3D printer: osteotomy templates, original mandible model, and titanium mesh

Then all designed templates of the patient’s mandible and osteotomy templates were saved as STL files and sent to a fully automated rapid stereolithography machine (SLA3500, 3D Systems, Texas, United States). They were printed using selective laser sintering (SLS) in polyamide, and the implant of titanium mesh was also printed (M2 cusing Multilaser, Concept Laser, German) (Fig. 3).

### Surgical procedure

**Fig. 4:**
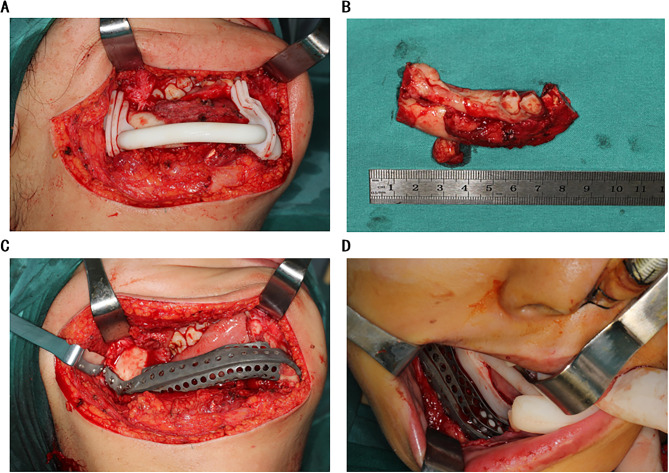
Resection of tumor and installation of titanium mesh. (A) The osteotomy templates were mounted. (B) The tumor was resected. (C, D) Titanium mesh after the installation was displayed in different angles

**Fig. 5:**
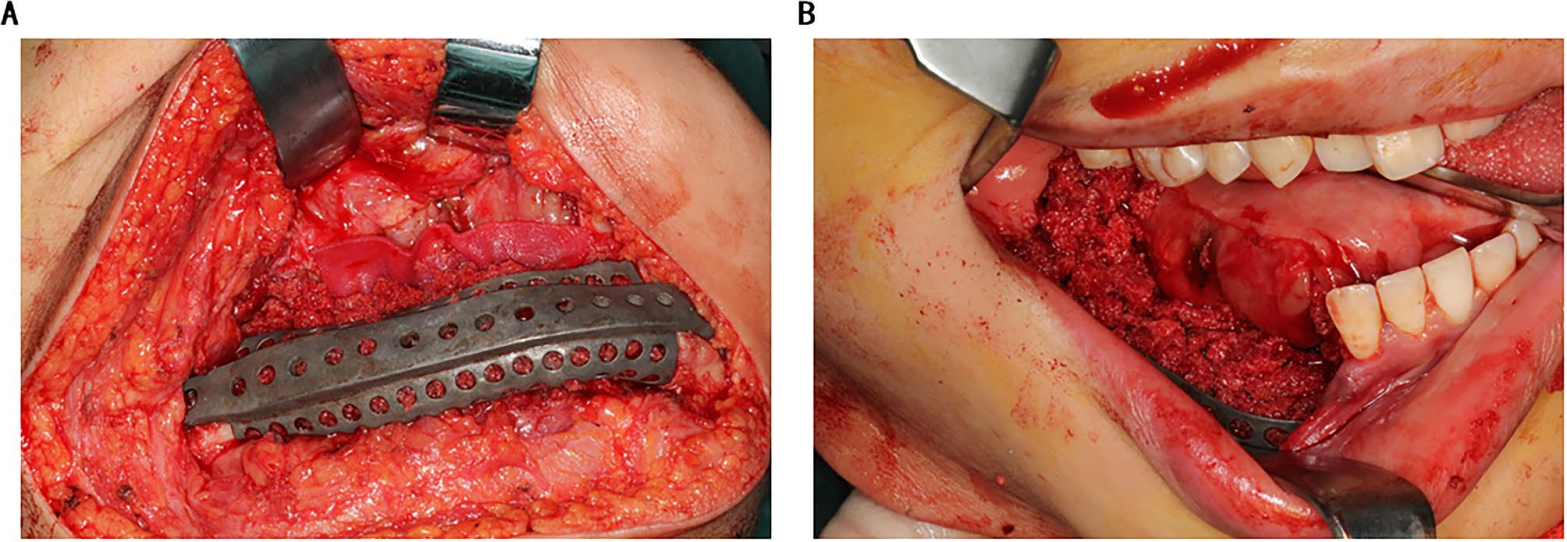
The harvest and placement of cancellous bone from the iliac bone

According to the surgical plan, the surgery was performed through the approach of inferior border of the mandible. The osteotomy templates were mounted on the buccal and inferior border of mandible. According to the templates, mandibular ameloblastoma was resected (Fig. 4A, B). The 3D-printed titanium mesh was installed with the predrilled hole method (Fig. 4C, D). Then cancellous bone graft was harvested from the right posterior iliac crest, and was filled into the titanium mesh (Fig. 5). Finally, the incisions in oral and submandibular region were tightly sutured.

### Evaluation

In this study, the accuracy of osteotomy with the guidance of 3D printed osteotomy templates, the symmetry of patient’s mandibular contour after surgery, and the resorption rate of the grafted bone 6 months after surgery were evaluated.

**Fig. 6:**
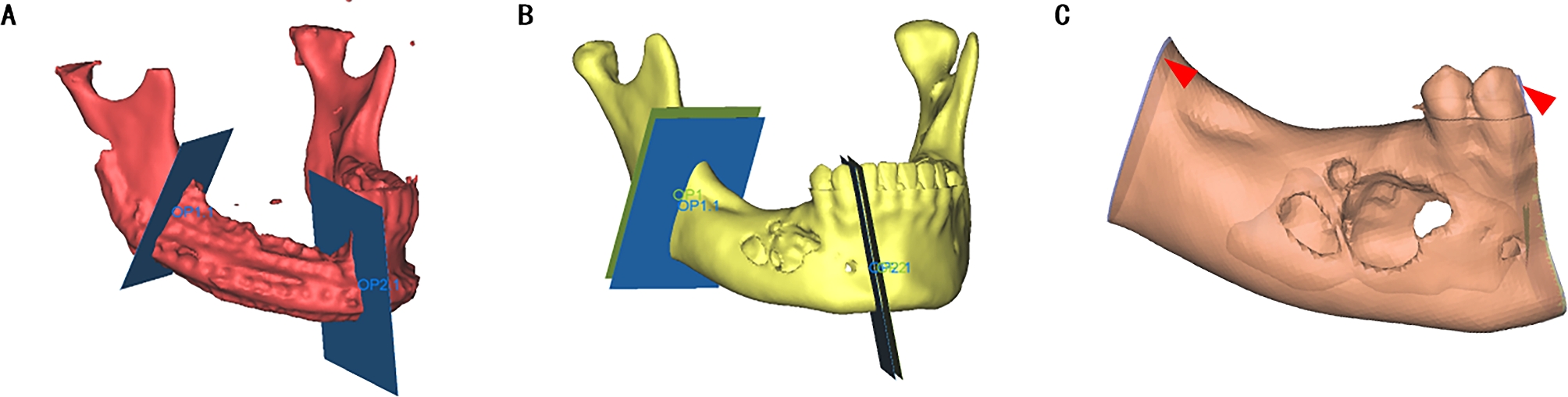
Volume of bone resection during surgery. (A) The osteotomy planes along the surgical incision were created. (B) The osteotomy planes were then superimposed on the preoperative mandible model. The blue plane is the bone resection plane during surgery, while the green plane is the bone resection plane in digital planning. (C) The block of bone resection during surgery was shown, and different areas from the digital planning are indicated by red arrows

**Fig. 7:**
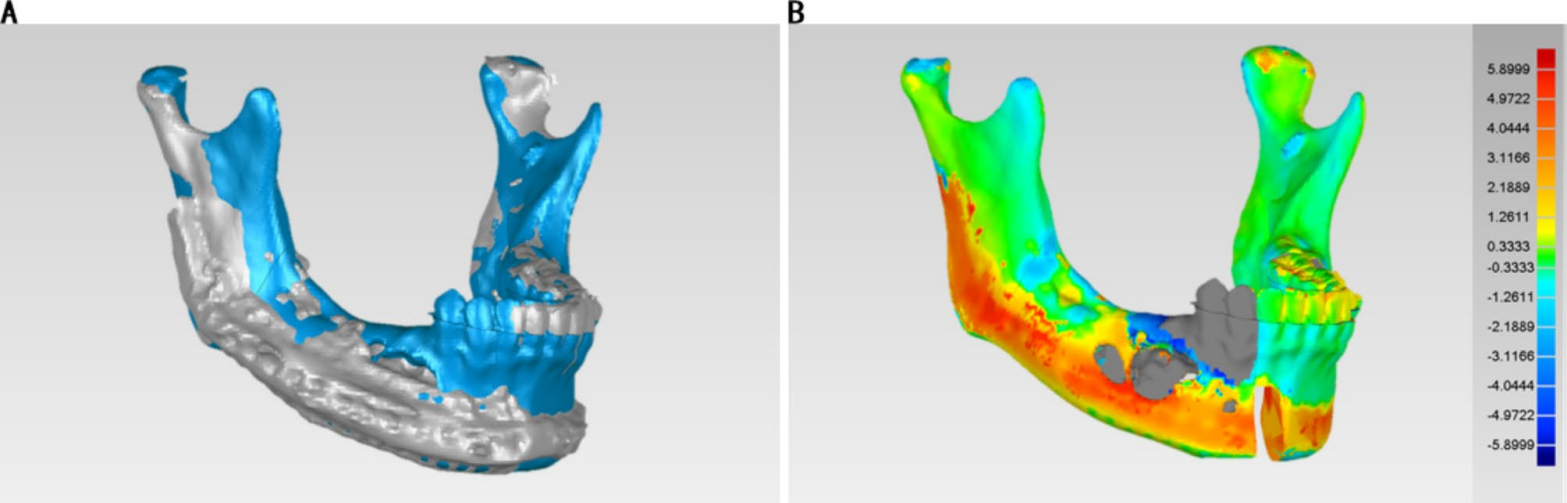
Discrepancy analysis of dental alignment between simulation and real result. (A) The 3D models of the patient’s preoperative mandible (blue) and mandible at 7 days postoperatively (gray) were imported into Geomagic Studio 2013 Software for alignment. (B) Discrepancy analysis of alignment between preoperative and postoperative mandible

To assess the surgical accuracy between digital planning and the actual outcome, image fusion of two models was performed. All superimposition and reference point determination processes were performed respectively using ProPlan software, in the “Scan registration wizard” of the “Segment” module and the “Measure and Analysis” of the “CMF/Simulation” mode. In the reconstructed model from postoperative CT scans, new osteotomy planes along the surgical incision were created (Fig. 6A). These planes were then superimposed on the preoperative mandible model to obtain an approximation of the intraoperative bone volume (Fig. 6B). The difference between the real result and the surgical plan was used as an indicator to assess accuracy (Fig. 6C). Geomagic Studio 2013 software allowed us to perform a 3D surface-to-surface matching process, which utilizes a least-mean-squared algorithm to align the actual postoperative mandible with the virtual surgical design and the deviations were measured as mean 3D deviation. (Fig. 7).

Patients with mandibular defects will seek restoration of occlusal function after surgery, such as implant restoration. Therefore, the bone resorption rates in the grafted area also need attention. The preoperative and postoperative CT images were compared by measuring the volume of harvested graft (V0), and the volume of the bone grafted 6 months later (V6). And the bone resorption rates (RR) is calculated as: RR= (V0-V6)/V0*100%.

### Statistical analysis

Statistical analysis was performed using SPSS version 25 (IBM, Chicago, IL, USA). The Kolmogorov-Smirnov and Shapiro-Wilk tests were used to assess the normality distribution. If the variable followed a normal distribution, a paired t-test was conducted. If not, the Wilcoxon signed-rank test was performed. Differences were considered statistically significant if the *p* was less than 0.05.

## Result

**Fig. 8:**
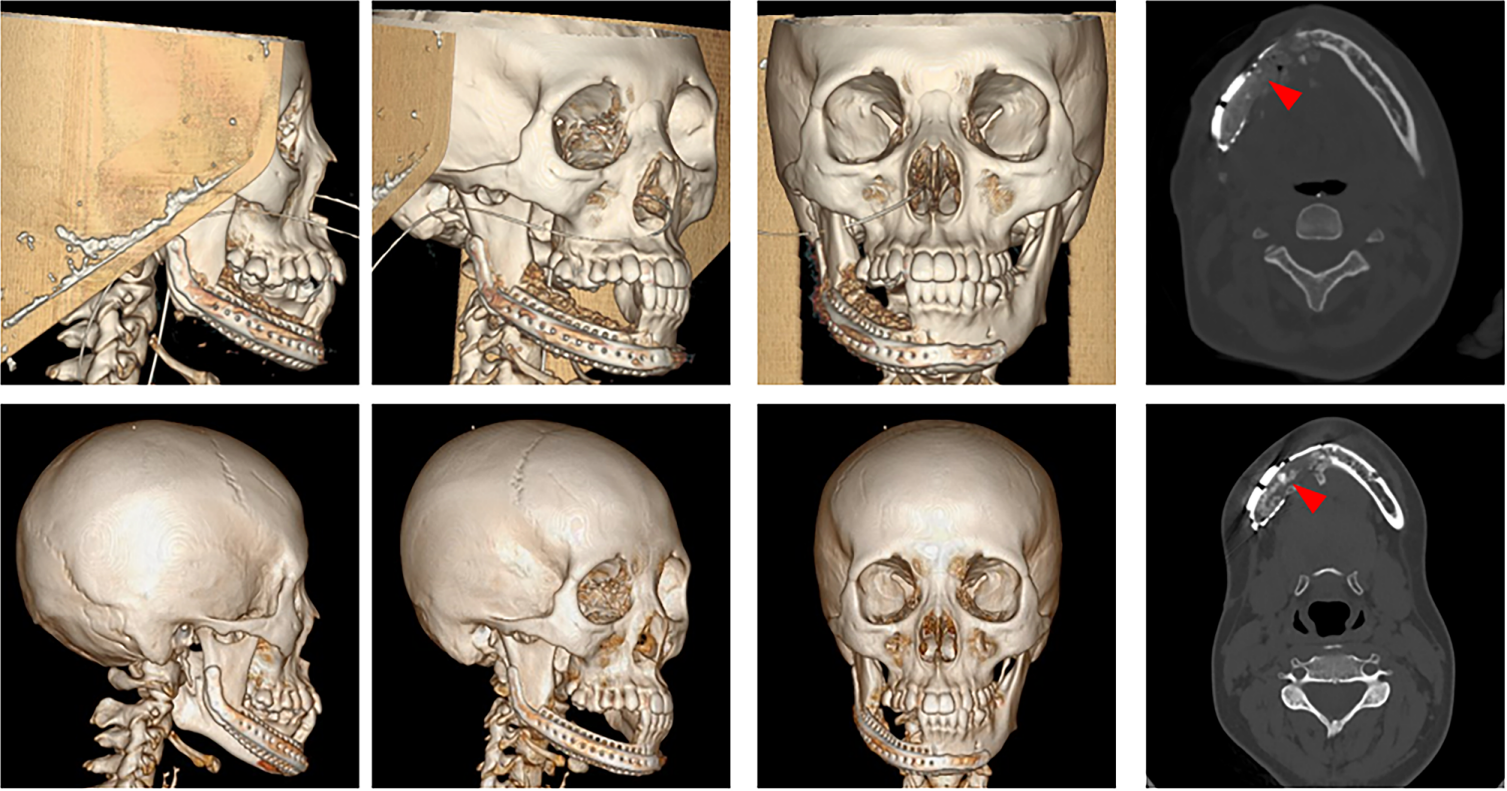
The CT images at seven days post-operation (upper) and six months post-operation (lower) Additional CT images taken at the same horizontal position were also displayed, with red arrows indicating the cancellous bone of the ilium

Using completely digital plans, all patients achieved satisfactory clinical outcomes without serious infections or complications. All patients underwent CT scans six months after surgery (Fig. 8) and no tumor recurrence occurred.

Combining the results of the model alignment, the postoperative contour of the patient’s mandible was consistent with the surgical plan except for a slight increase in the inferior border of the affected side. The mean 3D deviation of the mandible between the preoperative and postoperative periods was small. The discrepancy means between intraoperative bone volume and the digital planning was 2.44%±2.10%. And the mean resorption rate of the bone grafted 6 months later was 32.15%±6.95% (Table 1). There was no significant correlation between gender (p = 0.750) and age (p = 0.463).

**Table 1:** Accuracy of postoperative results compared with virtual surgical planning and bone resorption rates of the bone grafted

Sample	Gender/Age (years)	The mean 3D deviation (mm)	The volume of bone resection (mm^3^)			The volume of bone grafted (mm^3^)		
			Digital	Actual	Error (%)	V0	V6	RR
1	F/24	0.38	14136.63	14393.34	256.71 (1.82%)	18268.56	13463.15	26.30%
2	F/53	1.38	10817.81	11523.38	705.57 (6.52%)	13442.89	8612.43	35.93%
3	F/36	0.29	9949.12	10106.21	157.09 (1.58%)	14868.98	9106.65	38.75%
4	M/57	1.31	16505.47	16328.01	-177.46 (-1.08%)	18008.49	14442.32	19.80%
5	M/27	0.52	8581.26	8838.15	256.89 (2.99%)	10736.37	6254.12	41.75%
6	M/42	0.64	14975.48	15299.27	323.79 (2.16%)	20195.46	13954.27	30.90%
7	M/33	0.97	18364.92	18932.22	567.3 (3.09%)	22201.93	15192.67	31.57%
Average	38.86 ± 11.61	0.78 ± 0.41	13332.96 ± 3361.21	13631.51 ± 3350.45	298.56 ± 264.20 (2.44%±2.10%)	16817.53 ± 3705.05	11575.09 ± 3245.14	32.15%±6.95%

V0, the volume of harvested graft; V6, the volume of the bone grafted 6 months later;

## Discussion

Based on the recent 2017 WHO classification, several types of ameloblastoma can be identified, including the traditional type (solid/multicystic – AMSMA), unicystic (AM-UA), and extraosseous/peripheral (AM-PA) [12]. It is imperative to acknowledge that these subtypes exhibit localized infiltrative behavior and harbor the propensity to metamorphose into malignant or quiescent forms (AM-MA) [13, 14]. Therefore, for AMSMA, a segmental resection with a margin of 1–2 cm has been favored [15]. However, the determination of the osteotomy line during traditional mandibular osteotomies is heavily reliant on the experience and judgment of the operating surgeon, which may result in inaccuracies and recurrence. In this study, utilization of preoperative digital planning coupled with 3D printed osteotomy guides demonstrated concordance with the preoperative design in terms of the volume of resected bone, thereby mitigating unnecessary loss of native mandibular tissue and minimizing the likelihood of neoplastic recurrence. This technology created favorable preconditions for the subsequent restoration of occlusal dynamics and dental anatomy.

Vascularized free bone flaps are the most recommended approach for reconstructing mandible and soft tissue defects secondary to tumor resection, including fibular free flap (FFF) or iliac crest flap (ICF) [16]. For autogenous bone grafts, the fibula is the preferred option for long bone or angle-to-angle jaw reconstructions, but for mandibular reconstructions, the iliac crest is deemed superior [17]. Nevertheless, both FFF and ICF procedures carry the risk of vascular embolism, flap necrosis, and postoperative complications in the donor area, which may reduce the patient’s quality of life. According to recent research, individuals who receive either FFF or ICF procedures may have reduced joint range of motion, sensory impairments in the donor site, loading pain, and limited movement following surgery [18–20]. In contrast, posterior iliac cancellous bone can be utilized as a donor site in maxillofacial reconstruction, especially when restoring alveolar height deficits [21]. It supplies more cancellous bone to restore the alveolar height of the affected area while minimizing alterations to the profile of the iliac bone [22]. A previous research study exploring the repair of alveolar defects showed a 48.91% resorption rate of iliac cancellous bone [23], whereas this study found the rate to be 32.15%, suggesting that iliac cancellous bone could be a viable option for bone defects repair.

The augmented volume of the grafted iliac cancellous bone in this study superseded the bone block volume extracted during preoperative virtual surgery (Table 1). Previous research suggests that compared to other frequently used autogenous bone donor regions, the iliac bone has lower bone density and a more porous bone structure [24]. This structural nuance potentially culminates in an expanded bone volume at the reconstructive site. Factors contributing to reduced graft volume include autoimmune-induced osteoclastic resorption [25] and alterations in bone density in the reconstruction area [26]. Bone resorption commonly correlates with chronic inflammation, M1 macrophages activation, increased generation of reactive oxygen species (ROS), and extended periods of inflammation while bone is being regenerated [27, 28]. Moreover, the grafted cancellous bone not only offers mechanical support but also a vast reservoir of bone marrow-derived mesenchymal stem cells (BMSCs) [29]. BMSCs’ migration and differentiation from bone marrow are essential in transforming the cancellous bone’s porous structure into a structurally compact form that progressively evolves into cortical bone.

Modern digital surgical techniques have revolutionized mandibular reconstruction, offering unprecedented accuracy and efficiency. However, current biomaterials are inadequate in bridging or filling the anatomic shape and structure of lost bone tissue, making them incapable of meeting surgical demands for larger critical-sized defects [30]. Nevertheless, 3D printing technologies in bone tissue engineering offer a revolutionary advancement in traditional treatments for large bone defects by overcoming these challenges [31]. Surgeons can calculate and analyze the size and volume of jaw defects via computer-aided design/computer-aided manufacturing (CAD/CAM), designing osteotomy lines using virtual surgery for patients with ameloblastoma. Among all metal materials, titanium and its alloys offer commendable biocompatibility, high strength-to-weight ratio, low modulus of elasticity, and exceptional corrosion resistance, making them suitable as scaffolds for bone growth and reconstructing significant bone defects. [32]. A 3D-printed titanium mesh should have adequate compressive capacity to avoid any probable collapse or displacement during bone defect restoration, ultimately providing appropriate space and mechanical support for new bone growth [33]. In this study, the mean 3D deviation between the preoperative VSP-designed mandible and the actual mandible at 7 days postoperatively was 0.78 ± 0.41 mm. Previous research studies have also shown that the accuracy of osteotomies is significantly higher when the VSP and 3D printed osteotomy guides are used together [34, 35]. This approach minimizes collateral tissue damage and enables precise positioning of 3D-printed titanium mesh implants, thereby improving postoperative facial symmetry [36]. Additionally, the favorable metabolism of bone marrow mesenchymal stem cells, growth factors, and other substances that promote osteogenesis require sufficient blood supply [37]. The optimal osteogenic environment facilitated by adequate vascular supply makes titanium-based 3D-printed implants particularly advantageous.

According to this study, the digital surgical planning and 3D-printed templates facilitate surgical precision. Deviations between the virtual and actual outcomes were within acceptable margins. Surgeons involved in the planning and templating phases reported enhanced procedural familiarity and confidence. Despite these promising results, it’s noteworthy that discrepancies in volumetric and linear measurements still persist. These could emanate from multiple variables including residual surgical errors, minor distortions in CT scan models, inaccuracies in digital planning algorithms, and potential deformities in osteotomy templates.

## Conclusions

Above all, this study investigated a novel approach utilizing a fully digital treatment methodology for the reconstruction of bone defects resulting from ameloblastoma resection. Moreover, the digital workflow exhibited high levels of predictability, accuracy and effectiveness, ranging from pre-treatment assessment to final restoration.

## Data Availability

All data generated and/or analyzed during the current study are available from the corresponding author on reasonable request.
